# Monocyte Chemotactic Protein-1 as a Potential Biomarker for Early Anti-Thrombotic Therapy after Ischemic Stroke

**DOI:** 10.3390/ijms13078670

**Published:** 2012-07-12

**Authors:** Hans Worthmann, Reinhard Dengler, Helmut Schumacher, Andreas Schwartz, Wolfgang G. Eisert, Ralf Lichtinghagen, Karin Weissenborn

**Affiliations:** 1 Department of Neurology, Hannover Medical School, Carl-Neuberg-Str. 1, Hannover 30625, Germany; E-Mails: worthmann.hans@mh-hannover.de (H.W.); dengler.reinhard@mh-hannover.de (R.D.); eisert.wolfgang@mh-hannover.de (W.G.E.); 2 Boehringer Ingelheim Pharma GmbH & Co. KG, Binger Str. 173, Ingelheim 55216, Germany; E-Mail: helmut.schumacher@boehringer-ingelheim.com; 3 Department of Neurology, Nordstadt Klinikum Hannover, Haltenhoffstr. 41, Hannover 30167, Germany; E-Mail: andreas.schwartz@krh.eu; 4 Institute of Biophysics, University of Hannover, Herrenhäuserstr. 2, Hannover 30419, Germany; 5 Department of Clinical Chemistry, Hannover Medical School, Carl-Neuberg-Str. 1, Hannover 30625, Germany; E-Mail: lichtinghagen.ralf@mh-hannover.de

**Keywords:** ischemic stroke, monocyte chemoattractant protein-1 (MCP-1), antithrombotic therapy, neuroprotection, dipyridamole, acetylsalicylic acid (ASA)

## Abstract

Inflammation following ischemic brain injury is correlated with adverse outcome. Preclinical studies indicate that treatment with acetylsalicylic acid + extended-release dipyridamole (ASA + ER-DP) has anti-inflammatory and thereby neuroprotective effects by inhibition of monocyte chemotactic protein-1 (MCP-1) expression. We hypothesized that early treatment with ASA + ER-DP will reduce levels of MCP-1 also in patients with ischemic stroke. The EARLY trial randomized patients with ischemic stroke or TIA to either ASA + ER-DP treatment or ASA monotherapy within 24 h following the event. After 7 days, all patients were treated for up to 90 days with ASA + ER-DP. MCP-1 was determined from blood samples taken from 425 patients on admission and day 8. The change in MCP-1 from admission to day 8 did not differ between patients treated with ASA + ER-DP and ASA monotherapy (*p* > 0.05). Comparisons within MCP-1 baseline quartiles indicated that patients in the highest quartile (>217–973 pg/mL) showed improved outcome at 90 days if treated with ASA + ER-DP in comparison to treatment with ASA alone (*p* = 0.004). Our data does not provide any evidence that treatment with ASA + ER-DP lowers MCP-1 in acute stroke patients. However, MCP-1 may be a useful biomarker for deciding on early stroke therapy, as patients with high MCP-1 at baseline appear to benefit from early treatment with ASA + ER-DP.

## 1. Introduction

Considering recent findings in experimental animals and stroke patients, the extent of the secondary inflammatory response that immediately follows ischemic brain injury, is suggested to be significantly accountable for the outcome in ischemic stroke [1–5]. The chemokine monocyte chemotactic protein-1 (MCP-1) attracts leukocytes to ischemic tissue [6]. These activated leukocytes accumulate in the cerebral microvessels and trigger the inflammatory reaction by further release of cytokines, oxygen-free radicals and proteolytic enzymes like matrix-metalloproteinases [7].

Former preclinical and clinical studies have suggested anti-inflammatory properties of dipyridamole, such as scavenging of active oxygen metabolites, reduction of the TNF-alpha and IL-8 concentration and inhibition of the adhesion of neutrophils to vascular endothelium in acute ischemic stroke patients [8–13]. In cell cultures of human leukocytes and platelets the addition of dipyridamole inhibited the expression of MCP-1 [8].

We hypothesized that early treatment with ASA + ER-DP (acetylsalicylic acid + extended-release dipyridamole) in patients with ischemic stroke or TIA will result in reduced levels of MCP-1.

## 2. Results

### 2.1. Study Population

A total of 425 randomized patients (220 patients assigned to early ASA + ER-DP and 205 patients to early ASA alone) with TIA/stroke (median age 68 years; median NIHSS at admission 3) were included in this sub-study of the EARLY trial. Both treatment groups were well balanced except for a higher number of current smokers in the early ASA + ER-DP group (Table 1).

### 2.2. MCP-1 Levels after Differential Antithrombotic Treatment

The change in levels of MCP-1 from admission to day 8 did not differ between patients treated with ASA + ER-DP and ASA monotherapy as analyzed for the total study population (see Table 2, n.s. in Wilcoxon-Mann Whitney test).

### 2.3. Outcome at 90 Days Dependent on Stroke Severity but not on MCP-1

Changes of MCP-1 from admission to day 8 were not predictive for outcome at 90 days, whereas clinical symptoms at day 8 assessed by NIHSS had the strongest predictive value for outcome at day 90 assessed by mRS independent of treatment (*p* < 0.0001).

### 2.4. Association of MCP-1 Baseline Levels, Anti-Thrombotic Therapy and Outcome at 90 Days

**Figure 1:**
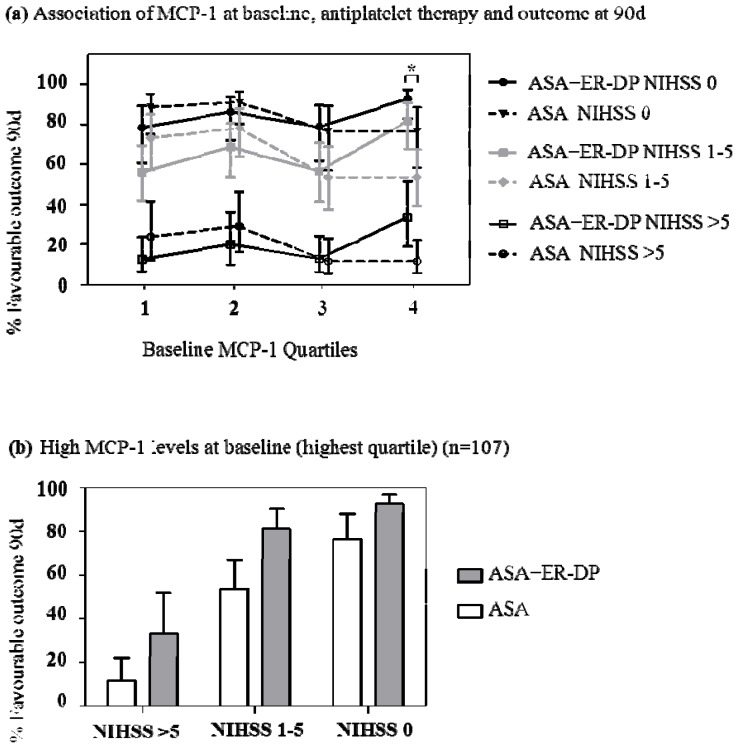
(**a**) (**b**) Association of MCP-1 baseline levels, anti-thrombotic therapy as either ASA + ER-DP (acetylsalicylic acid + extended-release dipyridamole) or ASA monotherapy and percentage of favorable outcome at 90 days. Patients in the highest MCP-1 quartile showed improved outcome if treated with ASA + ER-DP in comparison to treatment with ASA alone (*p* = 0.004 as indicated by *, Figure 1a). This effect was observed across all three NIHSS groups at baseline (Figure 1b). The predicted probability of favorable outcome as well as upper and lower 95% confidence limits are indicated. Baseline quartiles of MCP-1 (pg/mL): Q1: 50-≤ 145, Q2: >145-≤ 183, Q3: >183-≤ 217, Q4: >217–973.

Logistic regression analysis revealed a significant interaction between treatment and MCP-1 levels at baseline on favorable outcome at day 90 (*p* = 0.009), indicating that the effect of treatment was not consistent across all MCP-1 levels. Comparisons within MCP-1 baseline quartiles showed that patients in the highest quartile (>217–973 pg/mL) showed improved outcome at 90 days (mRS 0 and 1) if treated with ASA + ER-DP in comparison to treatment with ASA alone (*p* = 0.004) (Figure 1a). This effect was observed across all three NIHSS groups at baseline (Figure 1a,b). No significant differences between treatment groups were identified if MCP-1 levels were not in the uppermost quartile at baseline (in the first three quartiles the respective p-values were 0.093, 0.301 and 0.792).

## 3. Discussion

Acute ischemia triggers an inflammatory response which is more than a bystander. The early inflammatory response contributes to the secondary progression of the ischemic lesion by increased expression and release of cytokines and chemokines [1–33]. Thus, MCP-1 has been shown to be rapidly increased within hours after the event [3,14]. This study investigated the effect of ASA + ER-DP versus ASA monotherapy upon the inflammatory response marker MCP-1 in 425 ischemic stroke patients of the EARLY study.

*In vitro* experiments showed a direct inhibition of MCP-1 expression by dipyridamole either alone or in combination with ASA [8]. In contrast to this, our data did not show any differences for MCP-1 levels at day 8 or the change in MCP-1 levels from day 1 to day 8 between the treatment groups (Table 2). Nevertheless, our data showed that MCP-1 may be a useful biomarker for deciding on early stroke therapy as patients with MCP-1 levels in the uppermost baseline quartile did benefit more from early treatment with ASA + ER-DP compared to ASA monotherapy. Correlation of MCP-1 levels or change with clinical outcome was weak for lower MCP-1 plasma levels. Importantly, this effect seems to be independent of stroke severity at baseline (Figure 1a,b).

MCP-1 has repeatedly been suggested to play a pathogenic role in ischemic stroke. It has been considered as one of the key proteins for monocyte recruitment in inflammatory settings [6,15]. Smaller infarcts were observed for example after permanent middle cerebral artery occlusion in a mouse strain deficient in MCP-1 [16]. Recently Strecker *et al*. [17] suggested that smaller infarction in MCP-1-deficient mice might be caused by decreased inflammation since induction of IL-6, IL-1β and granulocyte-colony stimulating factor expression and subsequent influx of haematogenous cells was diminished. Additionally similar effects on reduction of infarct size and inflammatory cell infiltration have been observed in mice that were deficient for the MCP-1 receptor (CCR2) [18]. Since our data did not show any influence of ASA + ER-DP on MCP-1 levels, there is so far no argument that lowering of elevated MCP-1 levels might be responsible for amelioration of outcome after treatment with the combination therapy. However, treatment effects by ASA + ER-DP upon this inflammatory marker might have been missed due to study design, since follow-up of MCP-1 determination was not performed before day 8 after stroke.

The temporal course of MCP-1 levels after acute stroke onset shows a rapid increase within 6 h and a decrease thereafter [3]. In EARLY those patients with high baseline MCP-1 levels show a benefit from early treatment with ASA + ER-DP (Figure 1). High baseline MCP-1 levels in patients might identify a time point after acute stroke at which anti-inflammatory treatment is still effective. This hypothesis, however, cannot be proven since exact time of blood withdrawal was not noted in the case report.

Only further more detailed investigations will be able to shed light as to whether lowering of MCP-1 within the first hours after stroke onset further improves outcome. Other anti-inflammatory properties of dipyridamole might well also contribute to the observed improvement [8–13,19]. Pretreatment of cell cultures of human leukocytes and platelets with dipyridamole inhibited the expression of MCP-1 [8]. In brain endothelial cells dipyridamole attenuated MMP-9 levels after exposure to TNF-alpha or oxygen-glucose deprivation [20]. Dipyridamole inhibited the adhesion of neutrophils to vascular endothelium in acute ischemic stroke patients but not in chronic stroke patients or healthy controls [13]. In a rat model of middle cerebral artery occlusion administration of dipyridamole at reperfusion led to neurological improvement and reduction in infarct size. In this model MCP-1 levels in the infarcted hemisphere were not significantly decreased [21].

The interpretation of MCP-1 levels as a potential marker for decision making upon which of the currently available drugs should be used for early secondary stroke prevention is as yet over-conclusive. Importantly, these effects have only been observed in a subgroup of patients as defined by the inclusion criteria for EARLY [22]. But since recent clinical trials in acute stroke treatment besides the thrombolysis trials have failed to show beneficial effects, it has been discussed that due to heterogeneity in stroke patients subgroups need to be defined for treatment approaches. The current data might help to identify those patients that may benefit from a neuroprotective, anti-inflammatory effect of ASA + ER-DP treatment. However, considering that MCP-1 represents only one component of the inflammatory cascade after ischemic stroke the possible role of further mediators has to be elucidated. Moreover, further investigations are required that evaluate the influence of cardiovascular risk factors and comorbidities.

## 4. Experimental Section

### 4.1. Patients and Clinical Assessment

To prove the hypothesis MCP-1 was measured in blood samples from 425 of 543 patients of the EARLY trial taken on admission and day 8 [22]. EARLY was a prospective, randomized, open-label, blinded-endpoint trial, conducted between July 2007, and February 2009, in 46 stroke units in Germany that were certified according to national standards of the German Society of Stroke Medicine. At the start of the trial, patients aged 18 years or more who presented with symptoms of an acute ischemic stroke that caused a measurable neurological deficit (NIHSS score between 5 and 20) were eligible. Patients had to be randomized and treated within 24 h of stroke symptom onset. Because of slow recruitment during the first 4 months of the trial the inclusion criteria was modified to include patients with minor stroke (NIHSS score ≤20). Patients with intracranial haemorrhage identified on CT and those eligible for thrombolysis therapy were excluded. Further exclusion criteria are defined by the EARLY trial protocol [22].

### 4.2. Protocol Approval, Registration and Patient Consents

Patients gave written informed consent before participation in the trial. If patients were unable to sign the informed consent form they had to give verbal informed consent. The protocol was approved by the local institutional review boards and independent ethics committees. The trial was conducted in accordance with the Declaration of Helsinki and the Guideline for Good Clinical Practice [23,24]. Clinical Trial Registration: NCT00562588.

### 4.3. Procedures

Patients were randomly assigned (1:1) either to 25 mg ASA plus 200 mg extended-release dipyridamole orally twice daily within 24 h of symptom onset (early initiation) for 90 days, or to 100 mg ASA orally once daily for the first 7 days and 25 mg ASA plus 200 mg extended-release dipyridamole orally twice daily from day 8 to day 90 (late initiation). If necessary, study drugs were given via a nasogastric tube. Patients were allowed pre-treatment with antithrombotic agents before hospital admission and at hospital admission.

Tele-mRS was assessed at the Municipal Hospital Munich-Harlaching at days 8 and 90 by an independent expert as described for the EARLY Trial. Patients who died during the study period were assigned a mRS score of 6.

### 4.4. Measurement of MCP-1

MCP-1 was measured from peripheral venous blood samples (EDTA-plasma) taken from 425 patients on admission and day 8. MCP-1 was determined in EDTA-samples by enzyme-linked immunoabsorbent assay (ELISA) kits (R & D Systems). The interassay coefficient of variation was shown to be 7.0%. The intra-assay coefficient of variation was shown to be 7.8%.

### 4.5. Statistical Analysis

Statistical analysis was performed using the SAS statistical package, (*SAS*, version 8.2; SAS Institute: Cary, NC, USA, 2001). A total of 425 patients was included. Patients were stratified by clinical symptoms at baseline and day 8 using the NIHSS (minor symptoms: NIHSS 0, mild to moderate symptoms: NIHSS 1–5, severe symptoms: NIHSS > 5). Baseline values as well as changes within 8 days of MCP-1 were separated into quartiles. In a first model the predictive value of clinical symptoms at day 8 (minor, mild to moderate, severe) and changes in MCP-1 for the outcome at day 90 was analysed. A second analysis investigated the probability of a favorable outcome at day 90 (mRS 0 or 1) in dependency of baseline MCP-1 quartiles and treatment, adjusted for NIHSS at baseline. Both analyses were performed using logistic regression. The EARLY trial is registered, number NCT00562588.

## 5. Conclusions

In conclusion, the change in MCP-1 levels did not differ in patients treated within 24 h with ASA + ER-DP in comparison to treatment with ASA alone. However, patients with MCP-1 levels in the highest quartile at baseline showed improved outcome at 90 days if treated with ASA + ER-DP in comparison to treatment with ASA alone despite similar stroke severity at baseline. Further clinical studies are warranted to investigate the role of MCP-1 as a potential marker for deciding on early antithrombotic therapy.

## Figures and Tables

**Table 1:** Demographics and baseline characteristics.

	Total (*n* = 425)	Early ASA + ER-DP (*n* = 220)	Early ASA (*n* = 205)
Age (years) ≥ 65	68 (27–95)	67.0 (27–95)	69.0 (37–88)
	272 (64%)	131 (60%)	141 (69%)
Men	272 (64%)	146 (66%)	126 (62%)
White	424 (100%)	219 (100%)	205 (100%)
BMI (kg/m^2^)	27.4 (4.0)	27.4 (4.1)	27.3 (4.0)
BMI ≥ 30	103 (24%)	53 (24%)	50 (24%)

Smoking			

Never	198 (47%)	93 (42%)	105 (51%)
Ex-smoker	125 (29%)	64 (29%)	61 (30%)
Current	100 (24%)	63 (29%)	37 (18%)

Concomitant disease			

Hypertension	317 (75%)	161 (73%)	156 (76%)
Diabetes	102 (24%)	51 (23%)	51 (25%)
Hyperlipidaemia	142 (33%)	79 (36%)	63 (31%)
Atrial flutter or fibrillation	16 (4%)	9 (4%)	7 (3%)
Congestive heart failure	19 (5%)	6 (3%)	13 (6%)
History of prior stroke	61 (14%)	34 (16%)	27 (13%)
mRS	2 (0–5)	2 (0–5)	2 (0–5)
NIHSS	3 (0–20)	3 (0–15)	3 (0–20)

Data are median (range), *n* (%), or mean (SD). BMI = body mass index.

**Table 2:** Monocyte chemotactic protein-1 (MCP-1) at baseline and day 8.

	Total (*n* = 425)	Early ASA + ER-DP (*n* = 220)	Early ASA (*n* = 205)	*p*
Baseline	183 (145–217)	182 (143–215)	184 (148–223)	-
Day 8	186 (156–229)	186 (154–231)	186 (158–227)	-
Change from baseline	8 (−25–41)	9 (−21–41)	7 (−28–41)	n.s.

MCP-1 (pg/mL), data are median (interquartile range), Wilcoxon-Mann-Whitney test for changes from baseline.
